# What Comes First: Return to School or Return to Activity for Youth After Concussion? Maybe We Don't Have to Choose

**DOI:** 10.3389/fneur.2019.00792

**Published:** 2019-07-23

**Authors:** Carol A. DeMatteo, Sarah Randall, Chia-Yu A. Lin, Everett A. Claridge

**Affiliations:** ^1^School of Rehabilitation Science, McMaster University, Hamilton, ON, Canada; ^2^CanChild Centre for Disability Research, McMaster University, Hamilton, ON, Canada; ^3^ARiEAL, Centre for Advanced Research in Experimental and Applied Linguistics, McMaster University, Hamilton, ON, Canada

**Keywords:** children, adolescents, mild traumatic brain injury, concussion management, return to school, return to activity

## Abstract

**Objectives:** Return to School (RTS) and Return to Activity/Play (RTA) protocols are important in concussion management. Minimal evidence exists as to sequence and whether progression can occur simultaneously. Experts recommend that children/youth fully return to school before beginning RTA protocols. This study investigates recovery trajectories of children/youth while following RTA and RTS protocols simultaneously, with the following objectives: (1) to compare rates and patterns of progression through the stages of both protocols; (2) to evaluate symptom trajectories of youth post-concussion while progressing through stages of RTS and RTA; and (3) to propose a new model for concussion management in youth that involves the integration of Return to Activity and Return to School protocols.

**Methods:** In a 3-year prospective-cohort study of 139 children/youth aged 5–18 years with concussive injury, self-reported symptoms using PCSS and stage of protocols were evaluated every 48 h using electronic surveys until full return to school and activity/sport were attained. Information regarding school accommodation and achievement was collected.

**Results:** Sample mean age is 13 years, 46% male. Youth are returning to school with accommodations significantly quicker than RTA (*p* = 0.001). Significant negative correlations between total PCSS score and stage of RTS protocol were found at: 1-week (*r* = −0.376, *p* < 0.0001; *r* = −0.317, *p* = 0.0003), 1-month (*r* = −0.483, *p* < 0.0001; *r* = −0.555, *p* < 0.0001), and 3-months (*r* = −0.598, *p* < 0.0001; *r* = −0.617, *p* < 0.0001); indicating lower symptom scores correlated with higher guideline stages. Median full return to school time is 35 days with 21% of youth symptomatic at full return. Median return time to full sport competition is 38 days with 15% still symptomatic. Sixty-four percent of youth reported experiencing school problems during recovery and 30% at symptom resolution, with 31% reporting a drop in their grades during recovery and 18% at study completion.

**Conclusions:** Children/youth return to school faster than they return to play in spite of the self-reported, school-related symptoms they experience while moving through the protocols. Youth can progress simultaneously through the RTS and RTA protocols during stages 1–3. Considering the numbers of youth having school difficulties post-concussion, full contact sport, stage 6, of RTA, should be delayed until full and successful reintegration back to school has been achieved. In light of the huge variability in recovery, determining how to resume participation in activities despite ongoing symptoms is still the challenge for each individual child. There is much to be learned with further research needed in this area.

## Introduction

Concussion has become an epidemic in children and youth. The number of reported head injuries in Emergency Departments among youth playing sport has increased in the past decade by over 40% (1). The symptoms of concussions can often interfere with participation and performance in home, school, and community activities (2, 3). The current consensus for standard concussion management is the six-stage Berlin Return to Play recommendations (2). This statement and much of the literature now suggest a more conservative approach to the management of children/youth with concussion. It is, however, still unclear as to what “more conservative” entails. When they are symptomatic, children and youth are advised to rest for 48 h (4) then gradually resume regular activity with incremental increases in physical and cognitive activity within symptom tolerance (5, 6). Depression and anxiety may result as secondary sequelae if youth are socially isolated and removed from normal activity and participation for prolonged periods of time (7–10). Prolonged rest can lengthen recovery time and contribute to deconditioning (11, 12), therefore protocols for children must contain a balance of activity and rest to promote physical, emotional and cognitive recovery.

Both RTS and RTA protocols for pediatric concussion management should be conservative and individualized (2, 13–19). A number of protocols guiding families and youth through progressive recovery steps for safe Return to School (RTS) and Return to Activity/Play (RTA) have now been developed, are widely-accepted and are important aspects of pediatric concussion management (2, 20, 21).

Given that being a student is the primary occupation of childhood through to young adulthood, an emphasis on returning to school should be a top priority for children and families, more so than return to sport (22, 23). However, to date the emphasis in post-concussion management has been on return to play and sports (20, 21). A medical chart review showed that primary care physicians were providing return to school instructions to only 27.5% of patients as compared to return to sport instructions (51.6% of patients) (24). This imbalance may reflect the absence of research about post-concussive school issues (25, 26) and thus empirical evidence for specific methods and timelines for returning children to school is not yet available. An evaluation surrounding concussion education in Toronto schools found that 77% of responding schools have RTS protocols in place, in contrast to the 92% that had RTA protocols (27).

The CanChild Protocols for Concussion Management for children 5–18 years (Appendices 1, 2 in Supplementary Materials) (19, 28) were originally based on the Zurich return to play protocols and now the revised Berlin consensus recommendations (2). The CanChild protocols also have the same six stages of Return to Activity as the Zurich Return to Play recommendations, but they are more conservative in the way the child moves through the stages and have more detail about activities and intensity associated with each stage. Currently, there is minimal evidence supporting as to the sequence of RTS and RTA, whether they can be achieved simultaneously or whether RTS must be achieved before beginning RTA protocols. The focus now has shifted to the importance of return to school before return to activity/play. The Berlin Consensus (2) states that RTS must be completed before RTA begins, but this is ambiguous in clarifying whether that means fully back to school, or just starting to attend school. Others have suggested similar protocols. The Center for Disease Control and Prevention (29) recommends a gradual and cautious RTS, but does not mention how a RTS protocol can be integrated with a stepwise RTA protocol. Thomas et al. (11) noted that a majority of emergency department physicians instructed patients to rest for 1–2 days before returning to school whereas they instructed beginning a stepwise RTA protocol only after patients' symptoms resolved. Many Ontario school boards have adopted a similar approach, recommending full RTS prior to starting RTA protocols (30).

There is increased understanding, however, that youth may benefit from physical activity prior to complete symptom resolution, particularly among youth who are slow to recover (31–33). Moreover, a multisite, prospective cohort study by Canadian researchers indicates that youth who reported early physical activity post-injury (<7 days) have a 25% decreased risk in developing persistent post-concussive symptoms compared to youth who reported no early physical activity (34). These results suggest that early integration of a RTA protocol with RTS may lead to better health outcomes for youth with concussion.

The current scientific literature regarding concussion management has not adequately addressed how to integrate RTA and RTS protocols post-concussion in youth. Prior to doing so, it is important to understand the recovery trajectories of youth while following both RTS and RTA protocols.

Therefore, the objectives of this study were:

1. To compare rates and patterns of progression through the stages of both protocols;
2. To evaluate symptom trajectories of youth post-concussion while progressing through stages of RTS and RTA.
3. To propose a new model for concussion management in youth that involves the **integration** of Return to Activity and Return to School protocols.

## Methods

### Participants

Participants were recruited between November 2014 and December 2016 through the Emergency Department at McMaster Children's Hospital in Hamilton, community referrals from family health teams or sports medicine clinics. The inclusion criteria were as follows: (1) diagnosed with concussion by a physician within the last year; (2) between the ages of 5–18 years; and (3) *still symptomatic* at recruitment. Children and youth were excluded from the study if they had a confirmed significant brain injury requiring resuscitation, surgical intervention or admission to the pediatric critical care unit. Informed written consent was obtained from all parents and participants. This study was approved by the Hamilton Integrated Research Ethics Board in Hamilton, Canada.

### Procedures

These analyses are part of a larger prospective cohort study evaluating youth compliance to RTS and RTA concussion management protocols (35). Participating youth were monitored for up to 6-months post-recruitment. Upon enrollment to the study, the RTA and RTS protocols were explained to the participating youth and their parents by research staff including how to proceed through the stages and highlighting the importance of returning fully back to school before returning fully back to activity. The protocols were presented together, and youth were told to follow them both. Study data were collected and managed using REDCap electronic data capture software. While still symptomatic, youth were provided electronic questionnaires every 48 h where they were asked to report their current recovery stage within the RTA and RTS protocols, provide an indication of their level of cognitive and physical activity, and complete the Post-Concussive Symptom Scale (PCSS) (36). When enrolled in the study, participants were asked to record on the PCSS, how they were feeling 1-week prior to the injury. Being symptomatic was then based on the difference between the participants' identified pre-injury status and current reporting of symptoms. Upon symptom resolution, participants were asked to complete the same questionnaires biweekly until their final in-person assessment, which occurred 3-months post-symptom resolution.

Secondary outcomes, including reported school problems, grade changes, and use of school accommodations were also collected.

### Statistical Analysis

SAS version 9.4 and SPSS Statistics version 23.0 were used to conduct the data analyses. Demographic and injury information variables are presented with mean and standard deviation (SD), or median and lower and upper quartiles (Q1, Q3, respectively), when distributions are highly skewed.

A Spearman's rank order correlation analysis was used to investigate the relationship between a child's PCSS score and corresponding stage of RTS/RTA protocols, with a significance level being set as *p* < 0.02, as adjusted for the comparisons at the three-designated time-points. To approach this analysis comprehensively, three key time points were chosen: 1-week, 1-month and >1-month, because they represent the most commonly reported recovery times in the concussion literature (2, 37), and they reflect the symptom recovery strata in the CanChild concussion protocols.

*T*-tests were calculated to determine the mean differences in days to each stage during progress through RTS and RTA to completion. Statistical significance was set at *p* ≤ 0.05. All data were tested for normality using the Shapiro-Wilk test.

## Results

The characteristics of all participants are shown in Table 1. The final sample consisted of 139 children and youth aged 5–18 included 64 boys (46%) and 75 girls (53%) with a mean age of 13.4 years. Sport-related injury was the most prevalent cause of injury (74%). Of these injuries, 28% occurred during recreational play in gym class or at recess, 27% were hockey-related, and 14% were basketball-related.

**Table 1:** Participant characteristics.

	**All participants (*n* = 139)**	**<1 month recovery (*n* = 49)^a^**	**Slow to recover (>1 month) (*n* = 79)^a^**
**Age**, mean (SD) years	13.4 (2.87)	12.3 (2.72)	13.5 (2.79)
**Sex**, n (%)			
Males	64 (46.0)	26 (53.0)	29 (36.7)
Females	75 (53.9)	23 (46.9)	50 (63.2)
**Time from Injury at Recruitment**, median (Q1, Q3), days	7.8 (3.0, 33.0)	4.63 (1.82, 7.8)	19.9 (5.7, 75.5)
**Cause of Injury**, *n* (%)			
Sports-related	103 (74.1)	39 (79.5)	55 (69.6)
Fall	22 (15.8)	7 (14.2)	18 (22.7)
MVA	4 (3.0)	0 (0.0)	2 (2.5)
Other	10 (6.2)	3 (6.1)	4 (5.0)
**Number of Previous Concussions**, *n* (%)			
0	81 (58.3)	39 (79.5)	40 (50.6)
1–2	46 (33.3)	6 (12.2)	24 (30.3)
3+	12 (15.8)	4 (8.1)	15 (18.9)
**Total PCSS Score at baseline**, mean (SD)	40.1 (24.8)	30.7 (18.6)	43.4 (25.6)
**Stratum Information based on Symptom Resolution**, *n* (%)			
Symptom free within 1 month	49 (35.2)	–	–
Symptom free within 3 months	40 (35.2)	–	–
Symptoms last longer than 90 days	23 (16.5)	–	–
Never reached symptom resolution during study (i.e., 6 month follow-up)^a^	16 (11.5)	–	–

a *Missing data regarding symptom duration for 11 participants due to withdrawal from study or pending data collection*.

This was the first concussion for 58% of the participants. At recruitment, median time since injury was 7.8 days (with Q1 and Q3 being 3.0 days and 33.0 days, respectively) whereas the mean time since injury was 34.8 days with the minimum and maximum time being 2.9 h and 320.9 days, respectively. Fifty six percent were in the slow to recover group, that is symptoms persisting longer than 1-month. It should be noted that the recruitment sample purposely included a heterogenous sample of youth with possible time from injury any time within in 1 year post as long as they were still symptomatic. This was reflected in the large variability in symptom duration profiles.

**Figure 1:**
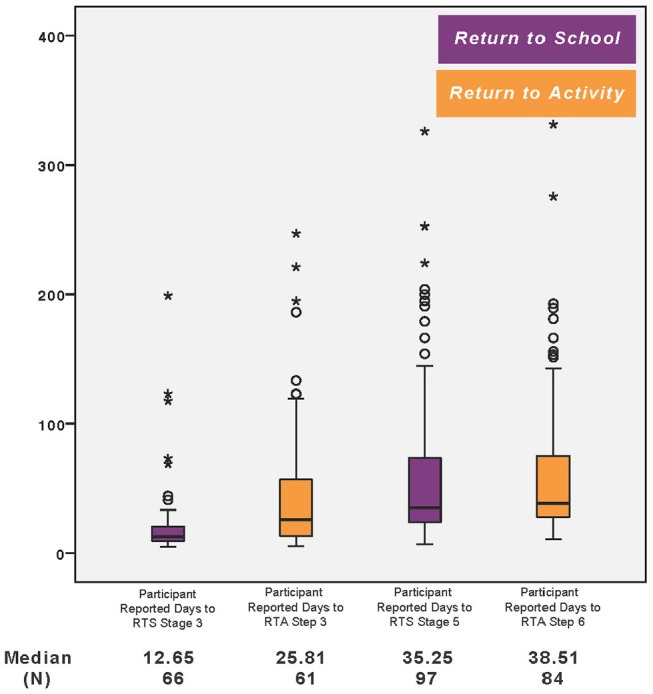
Participant reported days to RTA and RTS stages. The star represents the outliers for the participants in the slow to recover group (>1 month). The circle represents the outliers for the participants in the <1 month recovery group.

The median return time to stage 3 of RTA was 25.8 days and 12.6 days to RTS. The median time to RTA step 6, full return to activity or sports competition, was 38.5 days (Table 2) with a median return to school time of 35.3 days (*p* = 0.000) (Figure 1). The median time in stage 3 RTA was 0 days (mean 12.3 days) while median time in stage 3 of RTS was 12.6 days (mean 14.9) (Table 3). Table 4 shows the paired sample *t*-test for time to stages 3 and 5 and 6 for RTA and RTS.

**Table 2:** Parent and child comparison of time in days to return to school, return to activity, and reported school information.

	**All participants (*n* = 139)**	**<1 month recovery (*n* = 49)^a^**	**Slow to recover (*n* = 79)^a^**
**Symptom Duration^a^**, median (Q1, Q3), days	29 (18, 57)	18 (14, 22)	57 (41, 108)
**Days until Return to School**, median (Q1, Q3), days			
Parent Report	18.5 (7.25, 48.5)	13.6 (5.9, 22.6)	28.3 (8.4, 76.2)
Participant Self-Report **RTS Stage 3**—Modified Academics	12.6 (9.3, 21.3)	13.4 (9.81, 18.6)	18.2 (10.8, 39.1)
Participant Self-Report **RTS Stage 5**—Full Return to School	35.3 (23.4, 78.1)	24.6 (17.9, 30.2)	69.2 (41.1, 139)
**Days until Return to Activity**, median (Q1, Q3), days			
Parent Report	48.4 (30.6, 73.9)	34.3 (27.9, 49.9)	62.4 (51.2, 113)
Participant Self-Report **RTA Stage 3**—Individual Sport Specific Activity	25.8 (12.9, 59.7)	12.4 (8.89, 23.1)	47.2 (23.5, 102)
Participant Self-Report **RTA Stage 6**—Full Return to Activity/Sport	38.5 (27.9, 75.3)	29.2 (24.9, 33.9)	74.1 (47.3, 151)
**Reported School Problems**, *n* (%)			
During recovery	89 (64.0)	28 (57.1)	49 (62.0)
At symptom resolution	42 (30.2)	21 (42.8)	17 (21.5)
Unknown	10 (7.1)	-	13 (16.4)
**Reported School Accommodations**, *n* (%)			
Yes	108 (74.0)	35 (71.4)	58 (73.4)
No	25 (19.7)	14 (28.5)	10 (12.6)
Unknown	6 (6.3)	-	11 (13.9)
**Reported Drop in Grades**, *n* (%)			
Yes	43 (30.9)	1 (2.0)	20 (25.3)
No	41 (29.4)	23 (46.9)	22 (27.8)
Unknown	55 (39.5)	25 (54.9)	37 (46.8)

a *Missing data regarding symptom duration for 11 participants due to withdrawal from study or pending data collection*.

**Table 3:** Participant reported days in RTS and RTA stages.

**Participant group**	**Step**	***N***	**Mean (*SD*)**	**Median**	**Minimum**	**Maximum**
**RETURN TO SCHOOL STAGES**						
<7 days recovery	1	1	0.00	0.00	0.00	0.00
	2	2	3.00 (4.24)	3.00	0.00	6.00
	3	2	2.50 (3.54)	2.50	0.00	5.00
	4	2	3.50 (0.71)	3.50	3.00	4.00
	5	2	77.50 (6.36)	77.50	73.00	82.00
<1 month recovery	1	15	6.47 (8.98)	3.00	0.00	30.00
	2	32	3.88 (6.42)	0.00	0.00	24.00
	3	35	11.17 (9.51)	9.00	0.00	47.00
	4	38	8.84 (7.02)	6.00	0.00	29.00
	5	45	81.04 (40.08)	80.00	6.00	186.00
Slow to recover	1	33	14.76 (23.33)	6.00	0.00	111.00
	2	58	11.28 (23.30)	2.00	0.00	114.00
	3	64	58.25 (74.00)	33.50	0.00	405.00
	4	59	32.64 (44.22)	18.00	0.00	223.00
	5	55	93.69 (61.31)	92.00	0.00	353.00
**RETURN TO ACTIVITY STAGES**						
<7 days recovery	1	1	6.00	6.00	6.00	6.00
	2	2	2.50 (3.54)	2.50	0.00	5.00
	3	2	3.50 (0.71)	3.50	3.00	4.00
	4	2	0.00 (0.00)	0.00	0.00	0.00
	5	2	1.00 (1.41)	1.00	0.00	2.00
	6	2	76.50 (7.78)	76.50	71.00	82.00
<1 month recovery	1	15	5.47 (5.36)	3.00	0.00	16.00
	2	32	14.38 (8.02)	12.00	0.00	34.00
	3	35	5.69 (7.65)	2.00	0.00	24.00
	4	38	8.58 (12.39)	4.00	0.00	62.00
	5	45	2.27 (4.77)	0.00	0.00	21.00
	6	45	76.13 (42.30)	75.00	4.00	186.00
Slow to recover	1	33	15.91 (22.95)	9.00	0.00	96.00
	2	58	63.91 (84.97)	31.00	0.00	499.00
	3	64	26.66 (44.75)	11.50	0.00	278.00
	4	59	36.88 (58.74)	10.00	0.00	259.00
	5	55	12.75 (44.45)	0.00	0.00	326.00
	6	40	78.33 (43.07)	78.50	2.00	170.00

**Table 4:** Paired sample *t*-test for time to stages 3 and stages 5 & 6 for RTA and RTS.

		**Mean**	***SD***	***df***	**2-tailed sig**
Pair 1	Days to RTS Stage 3 & Days to RTA Step 3	27.1 41.6	37.2 49.9	43	*P* < 0.000
Pair 2	Days to RTS Stage 5 & Days to RTA Step 6	51.6 62.9	56.7 59.6	83	*P* < 0.000

Table 5 depicts the PCSS at stages 3 RTA/RTS and Stage 6 RTA and Step 5 RTS. The symptom score decreases over time, but symptom scores are consistently higher for RTS than RTA. Fifteen percent of participants were still reporting symptoms upon full return to activity, stage 6 RTA, while, 21% of participants were still symptomatic at stage 5 of the RTS protocols.

**Table 5:** Participant reported PCSS at RTA and RTS stages.

	**Participant reported PCSS at RTS stage 3**	**Participant reported PCSS at RTS stage 5**	**Participant reported PCSS at RTA step 3**	**Participant reported PCSS at RTA step 6**
*N*	66	97	60	84
Mean	14.89	5.71	12.27	3.17
Median	7	0.00	0.00	0.00
Minimum	0	0	0	0
Maximum	118	72	98	52
Percentiles 25	0.00	0.00	0.00	0
50	7.00	0.00	0.00	0
75	25.00	5.00	14.00	0

The Spearman's rank order correlation analysis showed significant negative associations between the total PCSS score and the stage of RTS/RTA protocols at 1-week (*r* = −0.376, *p* < 0.0001; *r* = −0.317, *p* = 0.0003), 1-month (*r* = −0.483, *p* < 0.0001; *r* = −0.555, *p* < 0.0001), and 3-months (*r* = −0.598, *p* < 0.0001; *r* = −0.617, *p* < 0.0001).

Sixty-four percent of youth reported experiencing school problems during recovery and 30% at symptom resolution with 31% reporting a drop in their school grades during recovery and 18% at study completion. Seventy four percent of parents reported their child was receiving school accommodations during recovery from concussion.

## Discussion

**Figure 2:**
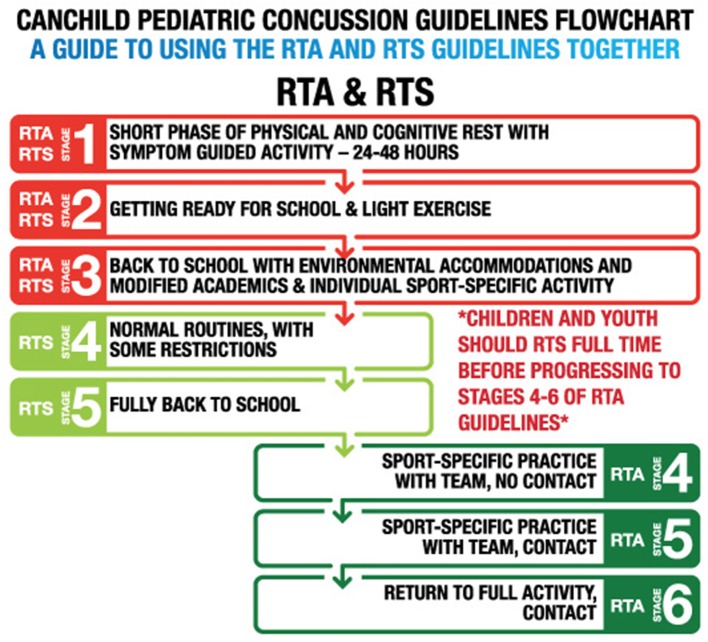
Return to activity and return to school protocol integration flowchart.

Days spent at each stage of recovery and time to attain each stage of recovery in Tables 2, 3 show that these times are comparable despite huge variability. The time difference between days to RTS and RTA are statistically significant with RTS being quicker. But overall the median time for both full return to school and full competition is just over 1 month. The trajectory of PCSS symptom scores decreases as stage increases, demonstrating a positive recovery trend as youth progress at their own pace through the stages of protocols showing continuous improvement without harmful effect or significant regression in stages or increase in symptoms while they follow RTS and RTA at the same time. Of note is that symptoms scores are higher in RTS stages and youth are more symptomatic on completing RTS (21%) than RTA (15%). This is understandable as youth are returning to school more quickly and not too concerning as long as they are receiving school accommodations, as seen in 74% of this study sample, so that they can continue with school while symptomatic, avoiding unnecessary academic failure and the resulting anxiety it produces for the youth. As expected, time to move through stages and time in each stage is quicker for the youth in strata one, recovery within 1 week, and strata 2, recovery within 1 month, with longest recovery times in the slow to recover group (Table 2) (38–40). The fact that youth return to school sooner than return to activity including contact sport is in line with the recommendations that youth should return to school before return to sport (2, 20, 41), except that in our study they are doing them in unison but at different rates. It is recommended that RTS stages should be followed in conjunction with the RTA protocol (17–19), as suggested in Figure 2. Stages 1–3 can be carried out simultaneously. After stages 1–3 there is more variability in youth recovery (Table 2) and more risk in the activity stages. It is suggested that RTS should then proceed up to full return to school attendance and performance, stage 5 RTS, before moving onto stage 4–6 in RTA. If a youth cannot participate fully in his or her academic program, then he or she should not be playing full contact or step 6 full activity and contact sport (2). As long as youth are active and participating, they do not have to go to full risk and contact sport (17–19, 42). In this way they can concentrate on getting back to full academic achievement while avoiding physical risk, though still enjoying and benefiting from some physical activity (43–46). We now know depression can affect youth who are not allowed to participate (7, 47). Therefore, the balance and compromise is to participate in both school and physical activity until higher level cognitive activity is required and higher level risk is present in higher stages of RTA protocol. At this point higher priority should be given to academic success (22) which is vital to future vocational opportunities and then proceed to higher risk physical activity as in stages 4–6 of RTA (2, 48).

The success of this approach is also greatly dependent on the school accommodations which we saw in 74% of youth. The accommodations allowed youth to progress successfully with both RTS and RTA. As presented in Table 2, the numbers of youth that have problems throughout recovery (64%) drops significantly by the end of symptom resolution (30%) and accommodations throughout by the school probably are likely a significant factor in this trend. It is interesting that quicker recovery of symptoms does not mean less problems as 43% of youth who recovered within a month had school problems as compared to 22% of those who had symptom resolution after 1-month post-injury. The fact that school problems decrease as recovery continues does not support the need to only complete school re-entry before activity resumption.

### Limitations

There are several limitations with this present study. There are inherent limitations with the self-report method and the current analyses were unable to confirm the accuracy of the self-reported symptoms and stage of guidelines. Missing data and those lost to follow-up and different numbers of youth completing each stage make analyses challenging. In addition, the huge variability in times to reach stages affects the normal distribution of the data, so the analyses had to be adjusted for this phenomenon. However, we deliberately chose to include youth with varying times post-injury as long as they were symptomatic in order that a realistic spectrum of recovery could be explored.

## Conclusions

Youth return to school faster than they return to play in spite of the self-reported, school-related symptoms they experience while moving through the protocols. Youth can progress simultaneously through the RTS and RTA protocols during the early stages 1–3. Considering the numbers of youth having school difficulties post-concussion, full contact sport, stage 6, of RTA, should be delayed until full and successful reintegration back to school has been achieved. In light of the huge variability in recovery, determining how to resume participation in activities despite ongoing symptoms is still the challenge for each individual child. There is much to be learned with further research needed in this area.

## Data Availability

The datasets generated for this study are available on request to the corresponding author.

## Ethics Statement

This study was carried out in accordance with the recommendations from the Hamilton Integrated Research Ethics Board with written informed consent and assent from all subjects. All subjects gave written informed consent in accordance with the Declaration of Helsinki. The protocol was approved by the Hamilton Integrated Ethics Research Board (HiREB).

## Author Contributions

CD contributed to study design and methodologies, data analysis and interpretation, manuscript preparation and critical revision, and final approval of the manuscript. SR contributed to data collection and analysis, manuscript development, critical revision, and final approval of the manuscript. C-YL contributed to data collection, data analysis and interpretation, manuscript development, critical revision, and final approval of the manuscript. EC contributed to data collection and analysis, manuscript development, and final approval of the manuscript.

### Conflict of Interest Statement

The authors declare that the research was conducted in the absence of any commercial or financial relationships that could be construed as a potential conflict of interest.

## References

[1] McFaull S Subaskaran J Branchard B Thompson W Emergency department surveillance of injuries and head injuries associated with baseball, football soccer and ice hockey, children and youth, ages 5-18 years, 2004 to 2014. Health Promot Chronic Dis Prev Can. (2016) 36:13–4. 10.24095/hpcdp.36.1.03 26789024 PMC4939465

[2] McCrory P Meeuwisse W Dvorák J Aubry M Bailes J Broglio S. Consensus statement on concussion in sport—the 5th international conference on concussion in sport held in Berlin, October 2016. Br J Sport Med. (2017) 51:838–47. 10.1136/bjsports-2017-097699 28446457

[3] Scorza KA Raleigh MF O'Connor FG. Current concepts in concussion: evaluation and management. Am Fam Phys. (2012) 85:123–32. Available online at: https://www.aafp.org/afp/2012/0115/p123.html 22335212

[4] Davis GA Anderson V Babl FE Gioia GA Giza CC Meehan Scolaro Moser R. What is the difference in concussion management in children as compared with adults? A systematic review. Br J Sports Med. (2017) 51:949–57. 10.1136/bjsports-2016-097415 28455361

[5] Haider MN Leddy JJ Pavlesen S Kluczynski M Baker JG Miecznikowski JC. A systematic review of criteria used to define recovery from sport-related concussion in youth athletes. Br J Sports Med. (2017) 52:1–14. 10.1136/bjsports-2016-096551 28735282 PMC5818323

[6] Wells EM Goodkin HP Griesbach GS. Challenges in determining the role of rest and exercise in the management of mild traumatic brain injury. J Child Neurol. (2015) 31:86–92. 10.1177/0883073815570152 25688071

[7] Stazyk K DeMatteo C Moll S Missiuna C Depression in youth recovering from concussions: correlates and predictors. Brain Injury. (2017) 31: 631–8. 10.1080/02699052.2017.1283533 28326857

[8] Berlin AA Deuster PA Kop WJ Autonomic nervous system activity as a predictor of fatigue following exercise withdrawal. Psychosom Med. (2006) 28:A–36. 10.1097/01.psy.0000204628.73273.23

[9] Guskiewicz KM Mihalik JP Shankar V Marshall SW Crowell DH Oliaro SM. Measurement in head impacts in collegiate football players: relationship between head impact biomechanics and acute clinical outcome after concussion. Neurosurgery. (2007) 61:1244–53. 10.1227/01.neu.0000306103.68635.1a 18162904

[10] Max JE Keatley E Wilde EA Bigler ED Schahar RJ Saunders AE. Depression in children and adolescents in the first 6 months after traumatic brain injury. Int J Dev Neurosci. (2012) 30:239–45. 10.1016/j.ijdevneu.2011.12.005 22197971 PMC3322312

[11] Thomas DG Apps JN Hoffman RG McCrea M Hammeke T Benefits of strict rest after acute concussion: a randomized control trial. Pediatrics. (2015) 135:213–23. 10.1542/peds.2014-0966 25560444

[12] Leddy JJ Kozlowski K Fung M Pendergast DR Willer B. Regulatory and Autoregulatory physiological dysfunction as a primary characteristic of post-concussion syndrome: implications for treatment. Neurorehabilitation. (2007) 22:199–205. 17917170

[13] Purcell LK Canadian Pediatric Society Healthy Active Living and Sports Medicine Committee Evaluation and management of children and adolescents with sport-related concussion. Paediatr Child Health. (2006) 11:420–8.

[14] Purcell LK. Evaluation and management of children and adolescents with sports-related concussion. Paediatr Child Health. (2012) 17:31–32. 10.1093/pch/17.1.31 23277754 PMC3276525

[15] Davis GA Purcell LK. The evaluation and management of acute concussion differs in young children. Br J Sports Med. (2014) 48:98–101. 10.1136/bjsports-2012-092132 23613516

[16] DeMatteo C Hanna SE Mahoney W Hollenberg RD Scott LA Law MC “My child doesn't have a brain injury, he only has a concussion.” understanding how clinicians use the word “concussion” in pediatric hospitals. Pediatrics. (2010) 125:327–34. 10.1542/peds.2008-2720 20083526

[17] DeMatteo C McCauley D Stazyk K Harper J Adamich J Randall S Post-concussion return to play and return to school protocols for children and youth: a scoping methodology. Disabil Rehabil. (2015) 37:1107–12. 10.3109/09638288.2014.952452 25144831

[18] DeMatteo C Stazyk K Singh S Giglia L Hollenberg R Malcolmson C. Development of a conservative protocol to return children and youth to activity following concussive injury. Clin Pediatr. (2015) 54:152–63. 10.1177/0009922814558256 25422524

[19] DeMatteo C Stazyk K Giglia L Mahoney W Singh SK Hollenberg R. A balanced protocol for return to school for children and youth following concussive injury. Clin Pediatr. (2015) 54:783–92. 10.1177/0009922814567305 25601958

[20] Halstead ME Walker KD Moffat K. Sport-related concussion in children and adolescents. Pediatrics. (2018) 142:e20183074. 10.1542/peds.2018-3074 30420472

[21] Lumba-Brown A Yeates KO Sarmiento K Breiding MJ Haegerich TM Gioia GA. Centers for disease control and prevention guideline on the diagnosis and management of mild traumatic brain injury among children. JAMA Pediatr. (2018) 172:e182853. 10.1001/jamapediatrics.2018.2853 30193284 PMC7006878

[22] Purcell LK Davis GA Gioia GA. What factors must be considered in ‘return to school' following concussion and what strategies or accommodations should be followed? A systematic review. Br J Sports Med. (2018) 53:250–65. 10.1136/bjsports-2017-097853 29500251

[23] Iverson GL Gioia GA. Returning to school following sport-related concussion. Phys Med Rehabil Clin N Am. (2016) 27:429–36. 10.1016/j.pmr.2015.12.002 27154854

[24] Arbogast K McGinley A Master C Grady M Robinson R Zonfrillo M. Cognitive rest and school-based recommendations following pediatric concussion: the need for primary care support tools. Clin Pediatr. (2013) 52:397–402. 10.1177/0009922813478160 23447397

[25] Halstead ME McAvoy K Devore CD Carl R Lee M Logan K. Returning to learning following a concussion. Pediatrics. (2013) 132:948–57. 10.1542/peds.2013-2867 24163302

[26] Snedden TR Pierpoint LA Currie DW Comstock RD Grubenhoff JA. Postconcussion academic support in children who attend a primary care follow-up visit after presenting to the Emergency Department. J Pediatr. (2019) 209:168–75. 10.1016/j.jpeds.2019.01.041 30853206

[27] Hacem LD Kourtis G Mylabathula S Tator C Experience with Canada's first policy on concussion education and management in schools. Can J Neurol Sci. (2016) 43:554–60. 10.1017/cjn.2016.41 27142787

[28] CanChild Centre for Childhood Disability Research Canchild Concussion Management - Return to Activity and Return to School Protocols. Hamilton, ON: CanChild, McMaster University (2019). Available online at: https://canchild.ca/en/diagnoses/brain-injury-concussion/brain-injury-resources (accessed February 21, 2019).

[29] Centre for Disease Control and Prevention HEADS UP to Schools: Know Your Concussion ABCs. U.S. Department of Health & Human Services (2015). Available online at: https://www.cdc.gov/headsup/schools/index.html (accessed February 12, 2019).

[30] Catholic District School Board. Concussion Protocol. Halton, ON (2016). Available online at: http://www.hcdsb.org/Parents/safeandhealthy/Medical/Pages/Concussion-Protocol.aspx (accessed February 12, 2019).

[31] Silverberg ND Iverson GL Grant L Is rest after concussion “the best medicine?”: recommendations for activity resumption following concussion in athletes, civilians, and military service members. J Head Trauma Rehabil. (2013) 28:250–9. 10.1097/HTR.0b013e31825ad658 22688215

[32] DeMatteo C Volterman KA Breithaupt PG Claridge EA Adamich J Timmons BW. Exertion testing in youth with mild traumatic brain injury/concussion. Med Sci Sports Exerc. (2015) 47:2283–90. 10.1249/MSS.0000000000000682 25871465

[33] Gauvin- LePage J Friedman D Grilli L Sufrategui M DeMatteo C Iverson GL. Effectiveness of an exercise-based active rehabilitation intervention for youth who are slow to recover after concussion. Clin J Sport Med. (2018). 10.1097/JSM.0000000000000634. [Epub ahead of print]. 30095507

[34] Grool AM Aglipay M Momoli F Meehan WP III Freedman SB Yeates KO Pediatric Emergency Research Canada (PERC) Concussion Team. Association between early participation in physical activity following acute concussion and persistent postconcussive symptoms in children and adolescents. JAMA. (2016) 316:2504–14. 10.1001/jama.2016.17396 27997652

[35] DeMatteo C Lin CY Foster G Giglia L Thebane L Claridge E Evaluating adherence to return to school and activity protocols in children following concussion. Clin J Sport Med. (2019). [Epub ahead of print]. 10.1097/JSM.0000000000000800 31876794

[36] Lovell MR Iverson GL Collins MW Podell K Johnston KM Pardini D. Measurement of symptoms following sports-related concussion: reliability and normative data for the post-concussion scale. Appl Neuropsychol. (2006) 13:166–74. 10.1207/s15324826an1303_4 17361669

[37] Ryu WH Feinstein A Colantonio A Streiner DL Dawson DR. Early identification and incidence of mild TBI in Ontario. Can J Neurol Sci. (2009) 36:429–35. 10.1017/S0317167100007745 19650352

[38] Barlow KM Crawford S Stevenson A Sandhu SS Belanger F Dewey D. Epidemiology of postconcussion syndrome in pediatric mild traumatic brain injury. Pediatrics. (2010) 126:e374–81. 10.1542/peds.2009-0925 20660554

[39] Leddy JJ Sandhu H Sodhi V Baker JG Willer B. Rehabilitation of concussion and post-concussion syndrome. Sports Health. (2012) 4:147–54. 10.1177/1941738111433673 23016082 PMC3435903

[40] Zemek R Barrowman N Freedman SB Gravel J Gagnon I McGahern C. Clinical risk score for persistent postconcussion symptoms among children with acute concussion in the ED. JAMA. (2016) 315:1014–25. 10.1001/jama.2016.1203 26954410

[41] Carson JD Lawrence DW Kraft SA Garel A Snow CL Chatterjee A. Premature return to play and return to learn after a sport-related concussion. Can Fam Phys. (2014) 60:e310, e312-5. 24925965 PMC4055342

[42] Lovell M Collins M Bradley J. Return to play following sports-related concussion. Clin Sports Med. (2004) 23:421–41. 10.1016/j.csm.2004.04.001 15262380

[43] Leddy JJ Hinds A Sirica D Willer B. The role of controlled exercise in concussion management. PM R. (2016) 8(Suppl. 3):S91–100. 10.1016/j.pmrj.2015.10.017 26972272

[44] Maerlender A Rieman W Lichtenstein J Condiracci C. Programmed physical exertion in recovery from sports-related concussion: a randomized pilot study. Dev Neuropsychol. (2015) 40:273–8. 10.1080/87565641.2015.1067706 26230745

[45] McLeod TCV Lewis JH Whelihan K Bacon CEW. Rest and return to activity after sport-related concussion: a systematic review of the literature. J Athl Train. (2017) 52:262–87. 10.4085/1052-6050-51.6.06 28387547 PMC5384824

[46] Schneider KJ Iverson GL Emery CA McCrory P Herring SA Meeuwisse WH. The effects of rest and treatment following sport-related concussion: a systematic review of the literature. Br J Sports Med. (2013) 47:304–7. 10.1136/bjsports-2013-092190 23479489

[47] DiFazio M Silverberg ND Kirkwood MW Bernier R Iverson GL. Prolonged activity restriction after concussion: are we worsening outcomes? Clin Pediatr. (2016) 55:443–51. 10.1177/0009922815589914 26130391

[48] Sady MD Vaughan CG Gioia GA. School and the concussed youth: Recommendations for concussion education and management. Phys Med Rehabil Clin N Am. (2011) 22:701–19. 10.1016/j.pmr.2011.08.008 22050944 PMC3208828

