# Supplementary material for: What Comes First: Return to School or Return to Activity for Youth After Concussion? Maybe We Don't Have to Choose
Source: Front Neurol. 2019 Jul 23;10:792. doi: 10.3389/fneur.2019.00792 (PMC6664873; doi:10.3389/fneur.2019.00792)
Supplement: Supplementary file 1 [file Data_Sheet_1.PDF]

RETURN TO SCHOOL GUIDELINES

STAGES 1-3 of the Return to Activity **(RTA)** and Return to School **(RTS)** guidelines should progress together, however youth should return full-time to school activities before progressing to STAGE 4, and 5 of the RTA guidelines

**STAGE 1**	**Short Phase of Physical and Cognitive Rest with Symptom Guided Activity 24-48 hours**	**GOAL:** NO SCHOOL for at least 24 hours. Home and leisure activities as tolerated, without an increase in the number or severity of symptoms. Notify school of concussive injury. NO physical activities of any intensity for longer than 5 minutes, as long as these activities do not increase symptoms.<br>**REST/LIGHT ACTIVITIES:** Regular daily activities that do not provoke symptoms such as self-care and easy tasks (e.g., making your bed, quiet socialization with a friend, talk on phone). LIMIT screen time (e.g. TV, video games, texting) and reading.<br>**WHEN TO MOVE TO STAGE TWO?** → When symptoms are not exacerbated by regular daily activities or have disappeared. If symptoms persist past 1 week, then progress to STAGE 2 cautiously.
**STAGE 2**	**Getting Ready to Go Back to School**	**GOAL:** Begin simple cognitive activity at home for a maximum of 30 minutes, without worsening symptoms. If symptoms worsen, reduce activity.<br>**ACTIVITIES:** Walking, 15 minutes of screen time/school work twice daily; socialize with 1-2 friends for no longer than 30 minutes.<br>**WHEN TO MOVE TO STAGE THREE?** → When symptoms have disappeared, decreased, or if symptoms persist past 2 weeks then move to STAGE 3 with support from school and medical professionals.
**STAGE 3**	**Back to School with Environmental Accommodations and Modified Academics**	**GOAL:** Build up your back-to-school routines by increasing cognitive activity in a school environment with accommodations. This stage may last days or months depending on the rate of recovery.<br>**ACADEMIC MODIFICATIONS are decided on an individual basis and guided by symptoms**<br>**TIMETABLE/ATTENDANCE:** Start by going for one hour, half days, or every other day. Try to reduce class time, later start time, or a shortened day.<br>**CURRICULUM:** Attend less stressful classes, allow more time to complete work, no tests, homework in 15-minute blocks for up to a maximum of 45 minutes daily.<br>**ENVIRONMENT:** Preferential seating, avoid computer, music, and gym classes, avoid noisy/crowded environments such as the cafeteria. Use headphones or sunglasses if sound or light sensitive. Provide a quiet work space or rest breaks during class.<br>**ACTIVITIES:** Limit screen/TV time into 15-minute blocks for up to 1 hour daily. General school activities: No school bus, lunch room, recess, and carrying heavy books.<br>**WHEN TO MOVE TO STAGE FOUR?** → When activities are tolerated without increasing symptoms.
**STAGE 4**	**Normal Routines, with Some Restrictions**	**GOAL:** Back to full days of school, but can do less than 5 days a week if needed due to fatigue or other continued symptoms.<br>**ACTIVITIES:** Complete as much homework as tolerated without causing or worsening symptoms. Only 1 test per week, may require shorter test or more time to complete. The student should NOT be required to catch up on missed work or exams in addition to new learning and curriculum.
**STAGE 5**	**Fully Back to School**	**GOAL:** Gradual return to normal routines including regular attendance, homework, tests, and extracurricular activities.

STOP

If symptoms increase or return at any STAGE, reduce activity by returning to the previous stage for 24 hours.

IMPORTANT NOTES

ANXIETY can be high after a brain injury. Many youth worry about school failure and need reassurance that accommodations will be temporary.

DEPRESSION is common during recovery from brain injury, especially when the child is unable to be active. Depression may make symptoms worse or prolong recovery.

Note: Different people recover at different rates depending on many factors, including severity of injury and previous health history. These timelines are meant to help set expectations and to be used as a guide. If you are worried about the pace of your recovery, contact a physician or brain injury specialist.

McMaster University

CanChild

For more information, please visit

www.canchild.ca

©CanChild, McMaster University 2018

CIHR IRSC
Canadian Institutes of Health Research
Instituts de recherche en santé du Canada
