# Supplementary material for: What Comes First: Return to School or Return to Activity for Youth After Concussion? Maybe We Don't Have to Choose
Source: Front Neurol. 2019 Jul 23;10:792. doi: 10.3389/fneur.2019.00792 (PMC6664873; doi:10.3389/fneur.2019.00792)
Supplement: Supplementary file 2 [file Data_Sheet_2.PDF]

RETURN TO ACTIVITY GUIDELINES

STAGES 1-3 of the Return to Activity **(RTA)** and Return to School **(RTS)** guidelines should progress together, however youth should return full-time to school activities before progressing to STAGE 4, and 5 of the RTA guidelines

STAGE 1 — Short Phase of Physical and Cognitive Rest with Symptom Guided Activity 24-48 hours

GOAL: Complete home and leisure activities as tolerated, without an increase in the number or severity of symptoms. No physical activities of any intensity for longer than 5 minutes, as long as these activities do not increase symptoms.

WHICH SYMPTOM GROUP ARE YOU IN? A B or C

A — SYMPTOM FREE WITHIN 48 HOURS AFTER INJURY	B — SYMPTOM FREE/SYMPTOMS DECREASE WITHIN 1-4 WEEKS AFTER INJURY	C — SYMPTOMATIC FOR MORE THAN 4 WEEKS
Take at least 24 hours for each stage as you complete the rest of the guidelines.	Take at least 2 days for each stage as you complete the rest of the guidelines.	Take at least 1 week for each stage as you complete the rest of the guidelines.

STAGE 2 — Light Exercise (No Contact)

Begin no later than 2 weeks post injury if symptoms are decreasing.
GOAL: In addition to activities accomplished in Stage 1, complete 15-30 minutes of light physical activity twice daily without worsening symptoms for a total of ~ 1 hour per day.
LIGHT ACTIVITIES: Walking, stationary cycling, swimming, stretching. NO resistance training or weight lifting.

STAGE 3 — Individual Sport-Specific Activity (No Contact)

GOAL: Able to complete activity requirements for Stages 1 and 2, with two additional 30-minute sessions of moderate physical activity daily without worsening symptoms for a total of ~ 2 hours per day.
MODERATE ACTIVITIES: Skating, light jogging, throwing.

STAGE 4 — Sport Specific Practice with Team (No Contact)

GOAL: Able to complete activity requirements for Stages 1-3, with two additional 30-minute sessions of moderate/vigorous physical activity for no more than 3 hours per day. By the end of the stage, progress to full team practice with NO CONTACT. Increase skill level and difficulty as tolerated without worsening symptoms.
MODERATE/VIGOROUS ACTIVITIES: Begin resistance training and general sport-specific conditioning skills with one other teammate.

STAGE 5 — Sport-Specific Practice with Team (Contact)

GOAL: Able to complete activity requirements for Stages 1-4, participate in full practice and training activities as tolerated without worsening or causing symptoms.
VIGOROUS ACTIVITIES: Running, cycling, jumping jacks.
If symptom free, you are ready to return to competition!

STAGE 6 — Return to Full Activity, Sport or Game Play (Contact)

Congratulations you have completed the Guidelines!

STOP

If symptoms increase or return at any STAGE, reduce activity by returning to the previous stage for 24 hours.

IMPORTANT NOTES

ANXIETY can be high after a brain injury. Many youth worry about school failure and need reassurance that accommodations will be temporary.

DEPRESSION is common during recovery from brain injury, especially when the child is unable to be active. Depression may make symptoms worse or prolong recovery.

Note: Different people recover at different rates depending on many factors, including severity of injury and previous health history. These timelines are meant to help set expectations and to be used as a guide. If you are worried about the pace of your recovery, contact a physician or brain injury specialist.

Reference: Berlin Consensus Statement on Concussion (2017)

For more information, please visit
www.canchild.ca

©CanChild, 2018
